# Supplementary material for: Ancient Jomon genome sequence analysis sheds light on migration patterns of early East Asian populations
Source: Commun Biol. 2020 Aug 25;3:437. doi: 10.1038/s42003-020-01162-2 (PMC7447786; doi:10.1038/s42003-020-01162-2)
Supplement: Supplementary file 1 — Supplementary Information [file 42003_2020_1162_MOESM1_ESM.pdf]

Supplementary Information

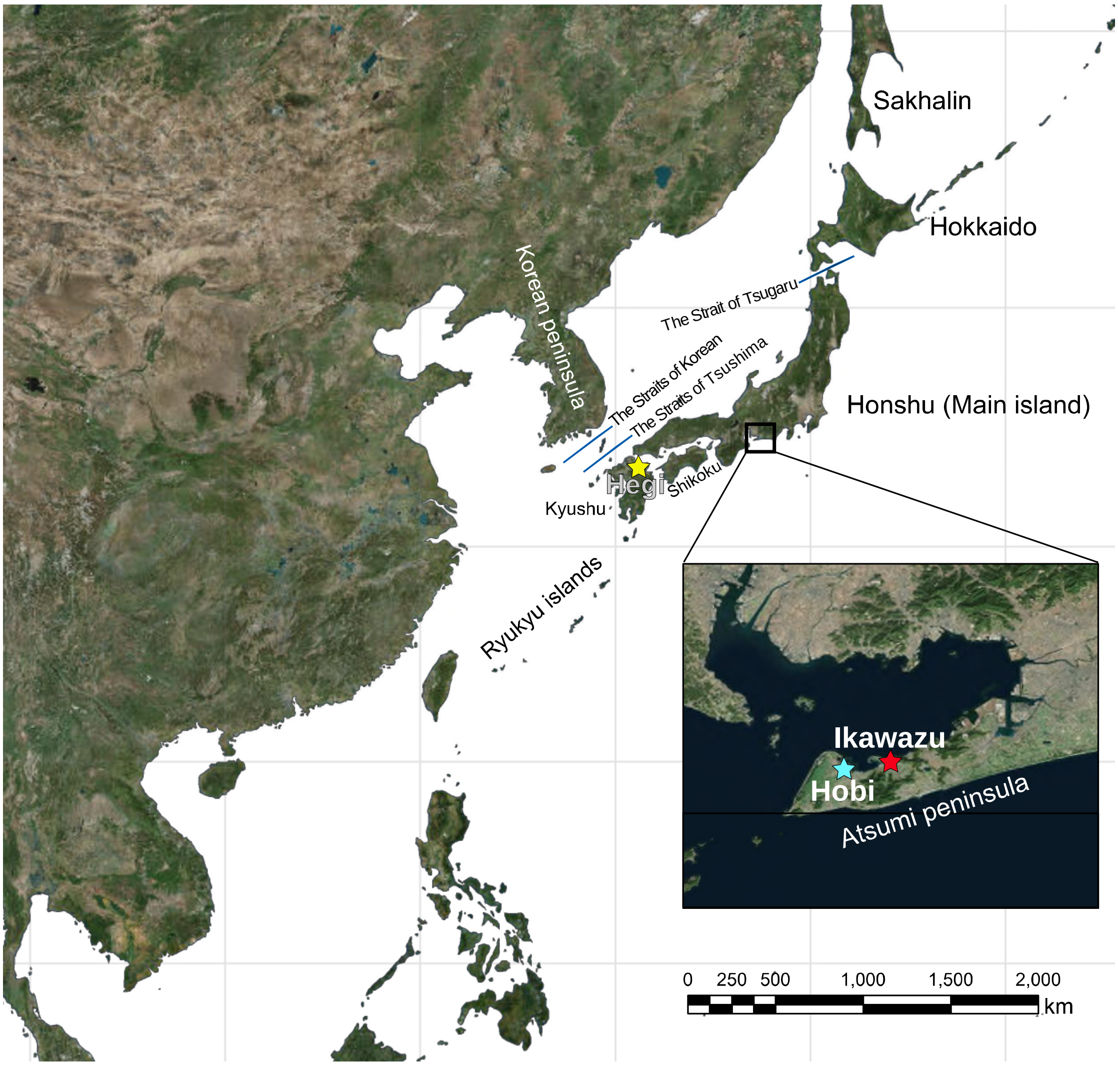

Supplementary Figure 1: Site location of Ikawazu shell-mound, Hobi shell-mound and Hegi cave in Japanese archipelago. The figure was made by ArcGIS 10.3 with the World Imagery map (Source: Esri, Maxar, GeoEye, Earthstar Geographics, CNES/Airbus DS, USDA FSA, USGS, AeroGRID, IGN, IGP, and the GIS User Community).

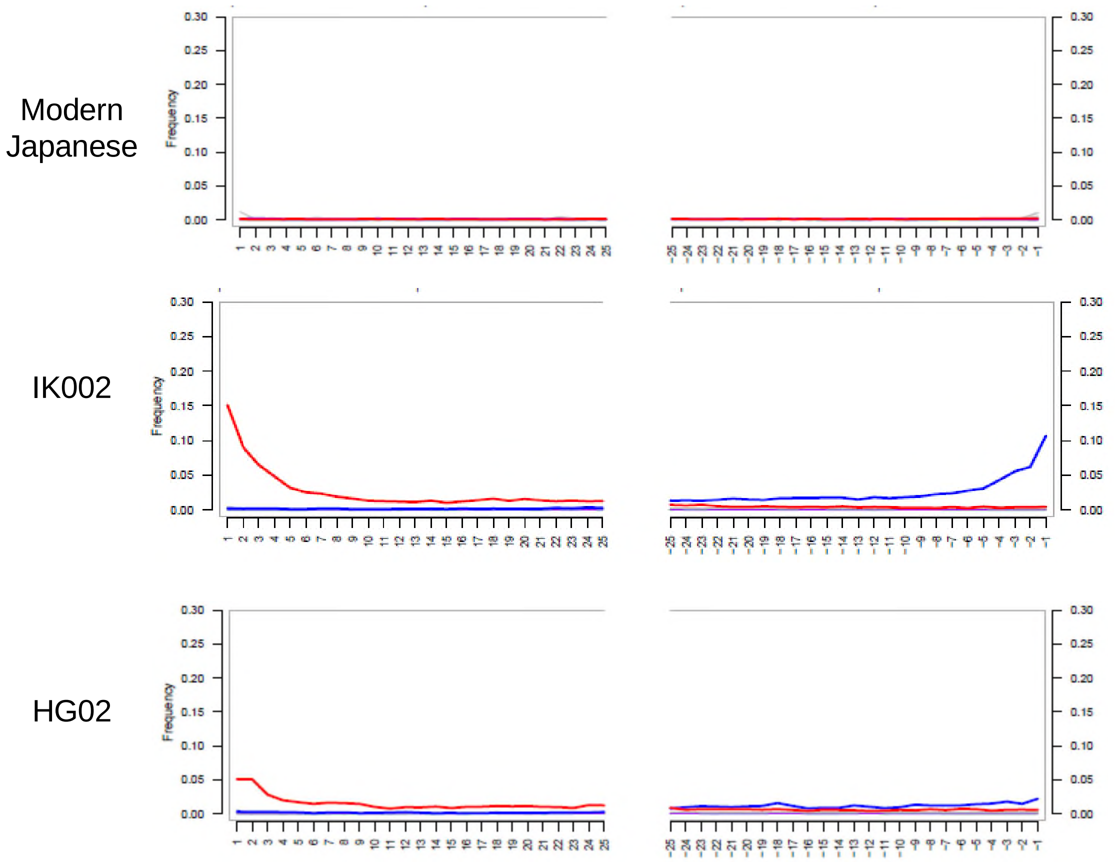

Supplementary Figure 2: Damage pattern of DNA molecules in a modern human, IK002 and Hegi02 (HG02) libraries constructed by modified protocols (NEBNext Ultra) in this study. The x-axis means nucleotide position from the end of 5’ and 3’, and the y-axis means the frequency of substitution for reference human genome (*hg19*). The Blue line represents C-to-T substitution pattern and the red line represents G-to-A substitution. Both frequencies in a modern blood sample is too low. On the other, the frequency of IK002 is clearly higher than that of the modern sample. The frequency of HG02 from the Hegi cave is relatively low than IK002.

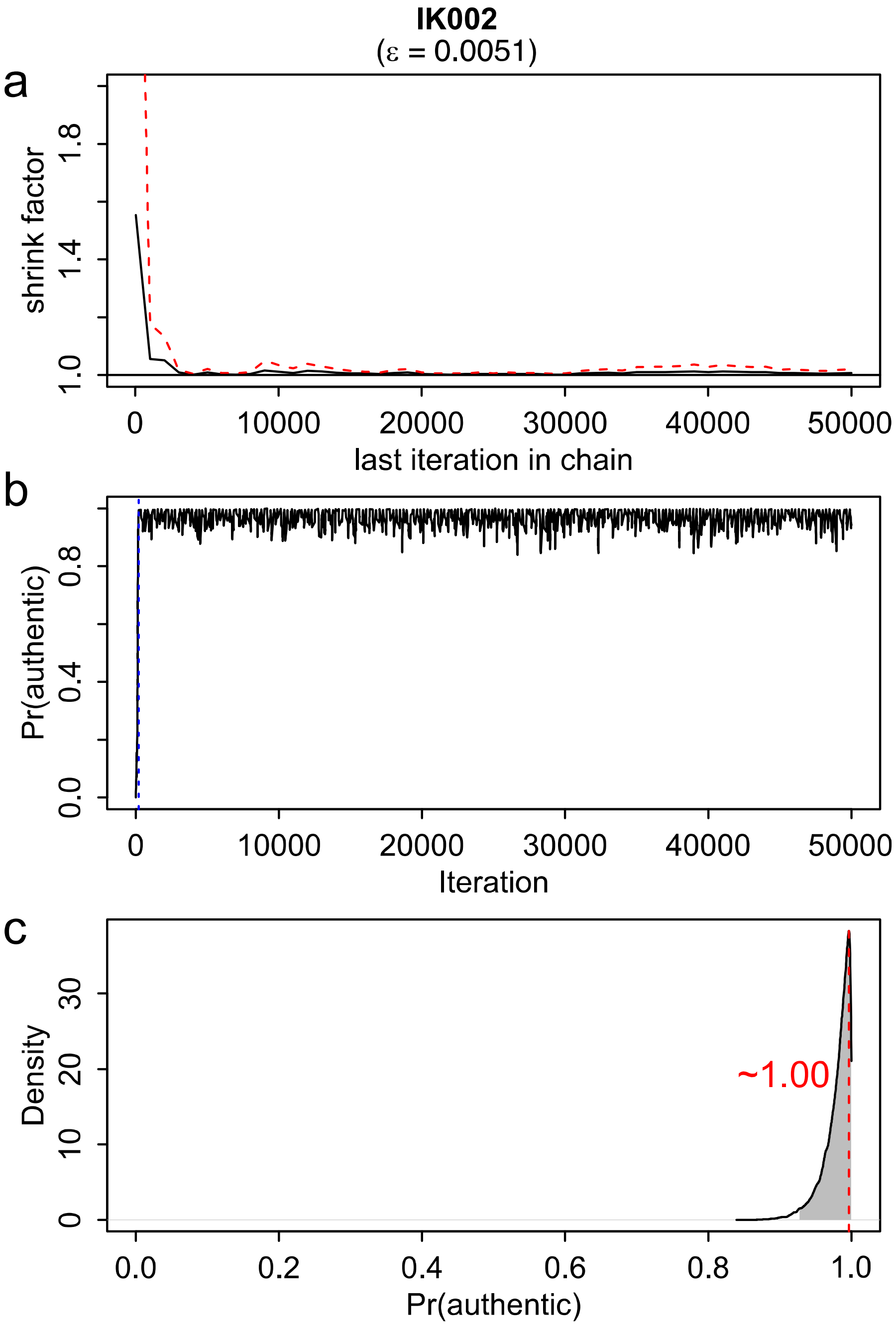

Supplementary Figure 3: Evaluating authentication of output data using maximum-likelihood of allele-mismatched patterns with mitochondrial DNA reads using *ContaMix*. **a.**The bootstrap iteration and the frequency of potential contamination risk using mitogenomic reads. The x-axis shows the number of bootstrap iteration and the y-axis shows the shrink factor. The *e* value means the contamination frequency between mitogenomic reads and reference sequences. **b.**The relationship between bootstrap iteration times (x-axis) and probability of estimating precision (y-axis). **c.**The relationship between the probability of estimation (x-axis) and the probability density (y-axis) calculated by the Bayesian estimation.

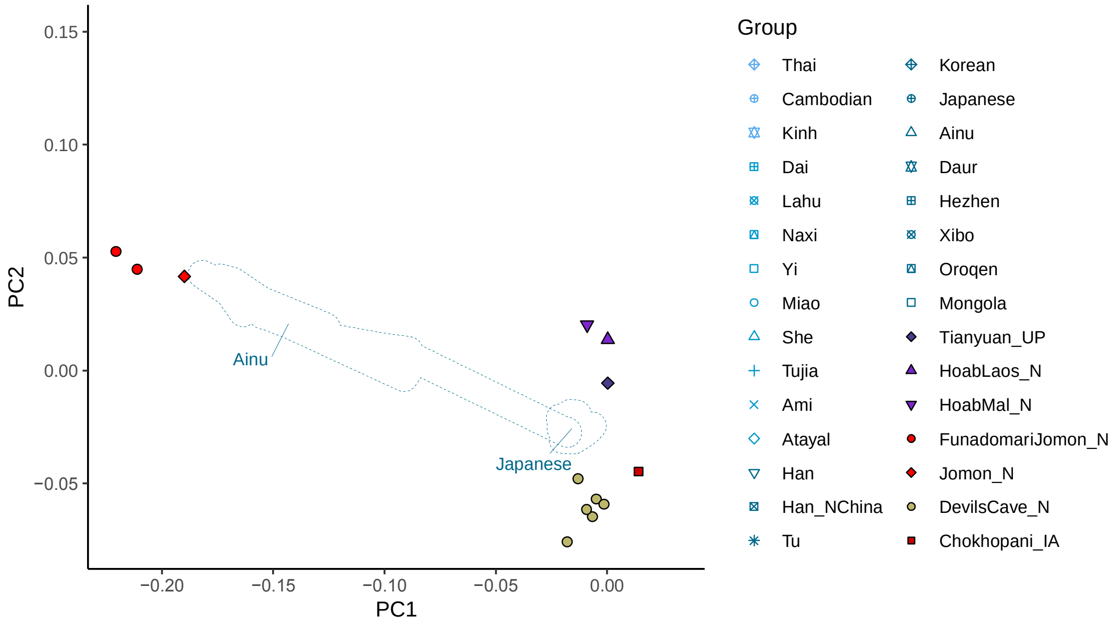

Supplementary Figure 4: PCA plot including Southeast, East and Northeast Asians, and ancient individuals (Ikawazu Jomon, Funadomari Jomon, Devil’s Gate Cave, Chokhopani, Hoabinian and Tianyuan).

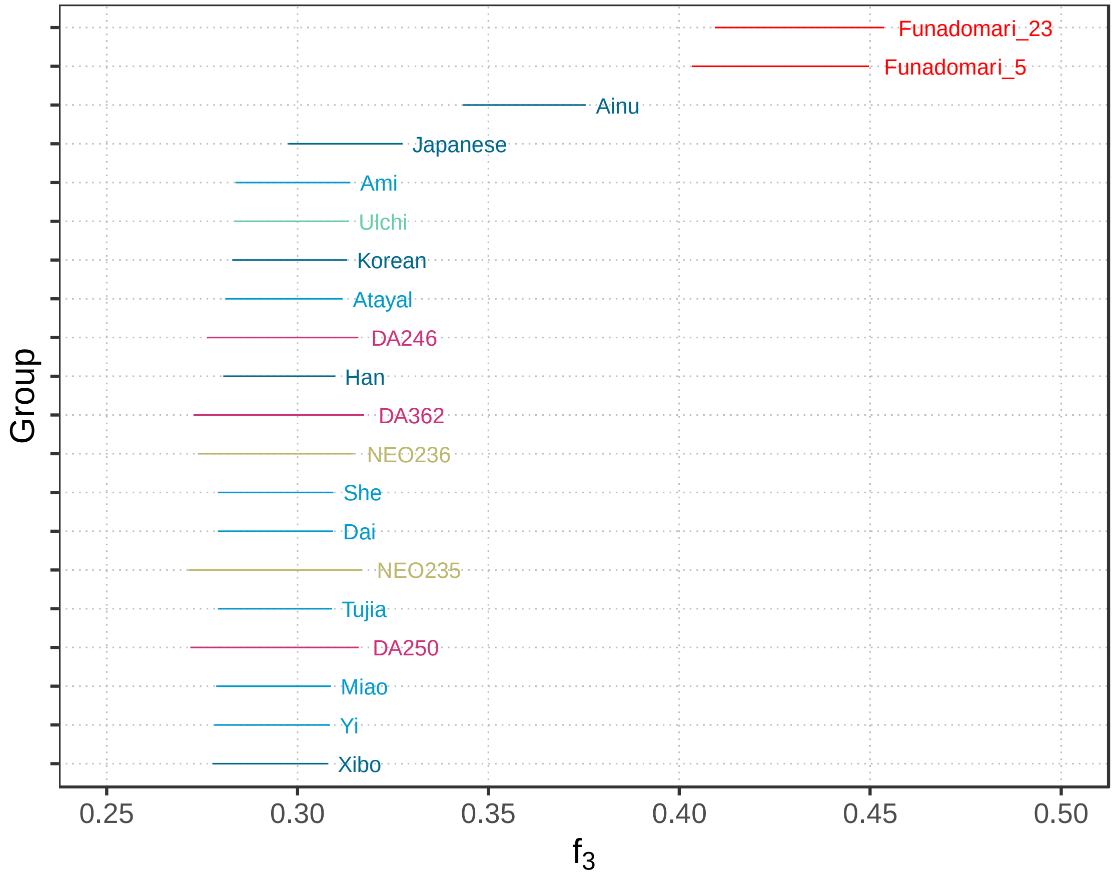

Supplementary Figure 5: Outgroup-f_3 statistics for IK002 with East Asians, Ainu and Hokkaido Jomons. Error bars indicate ± 3 s.e.

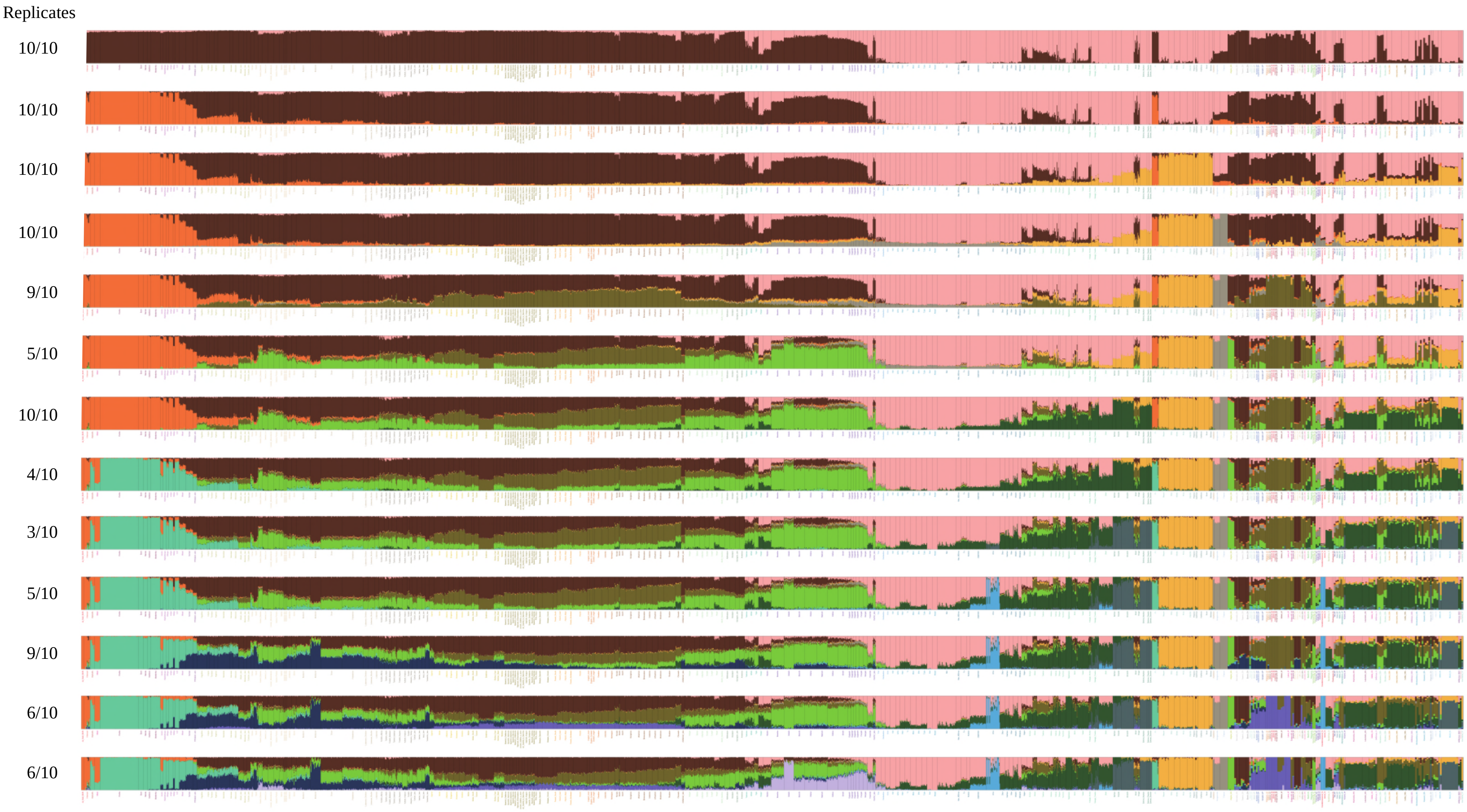
Replicates
10/10
10/10
10/10
10/10
9/10
5/10
10/10
4/10
3/10
5/10
9/10
6/10
6/10

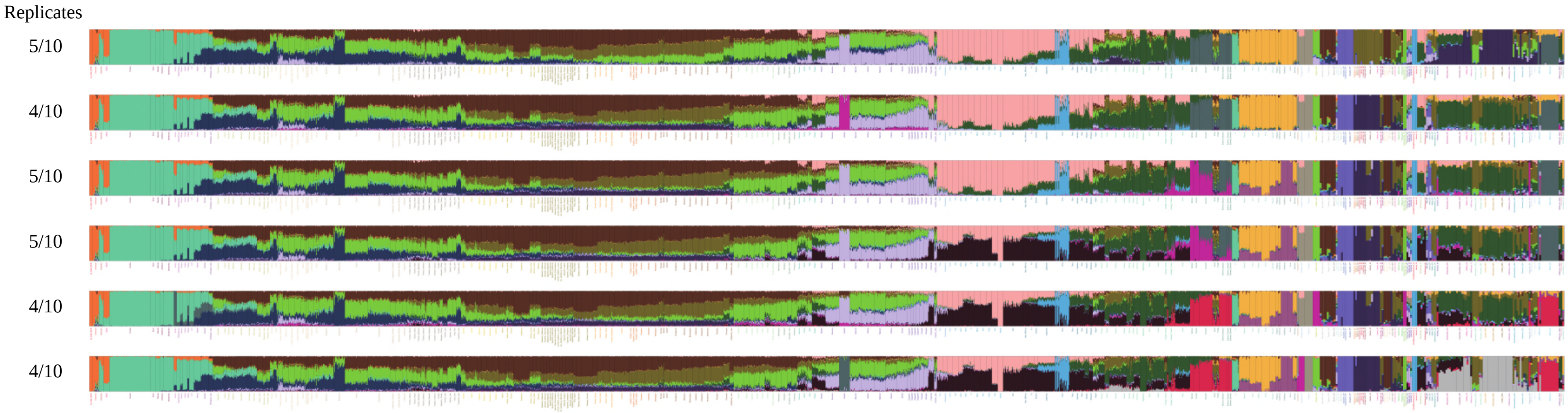

Supplementary Figure 6: ADMIXTURE ancestry components for ancient and contemporary individuals and ancient individuals. Each plot shows different K component (K=2~20) from top to bottom. Replicates show the number of iteration for running ADMIXTURE (denominator), and the selection number within each K plot (numerator). The x-axis represents individual data from different populations and the y-axis shows the frequency calculated by haplotype frequencies based on genome-wide SNPs. The color represents the unique component based on assumption of the number of cluster. The Jomon component (light blue color) was occurred from K=11.

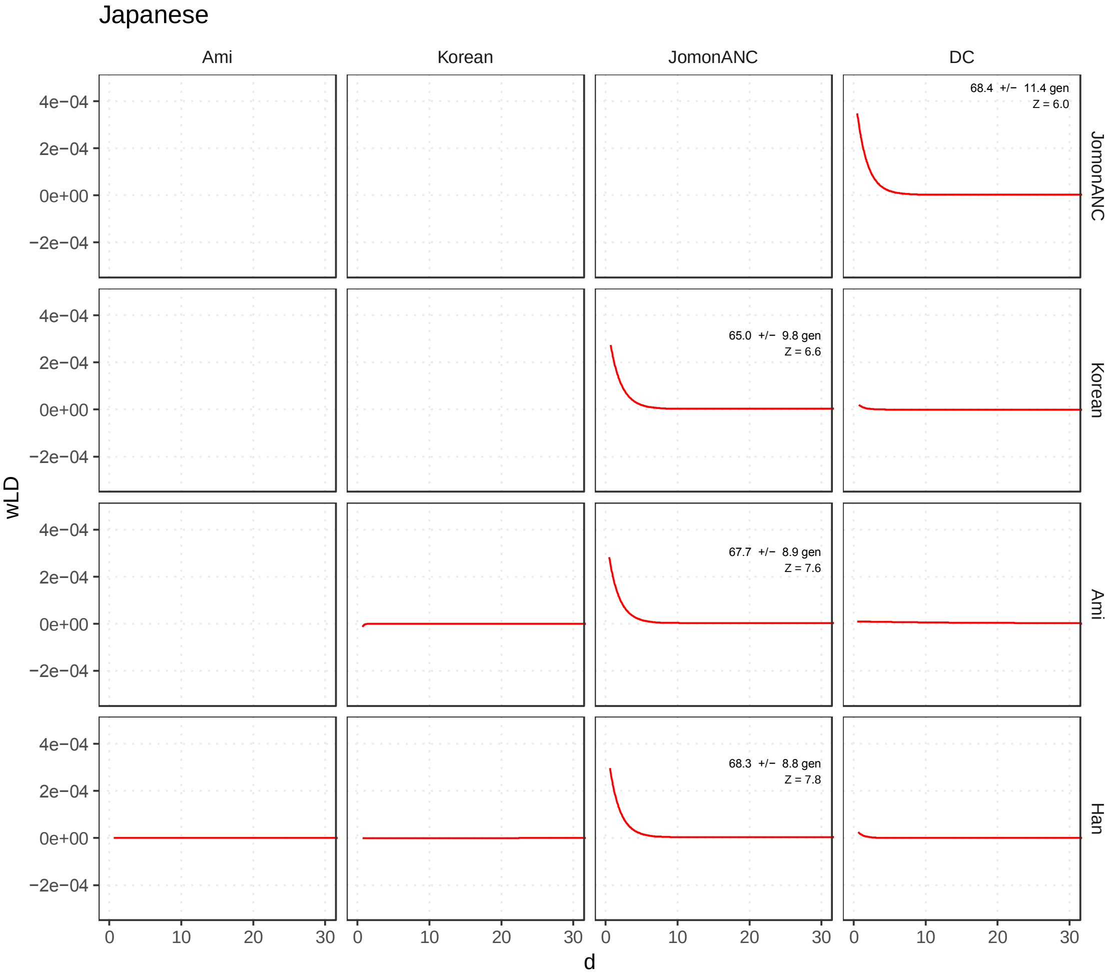

Supplementary Figure 7: Patterns of LD decay in admixture events of Japanese using ALDER with two source of ancestry populations (mainland East Asians (Korean, Ami, Han, Devils cave) and Jomon).

m = 1 , 97.28 % variance explained, ll = 1076.96

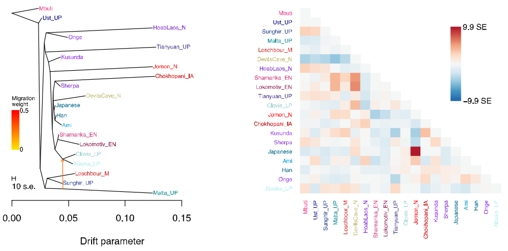

m = 2 , 98.07 % variance explained, ll = 1248.31

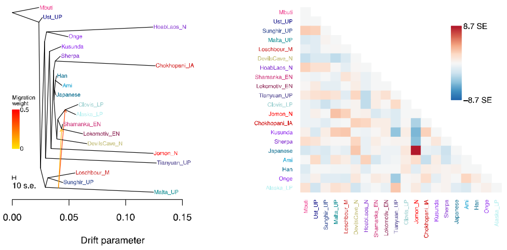

m = 3 , 98.42 % variance explained, ll = 1295.83

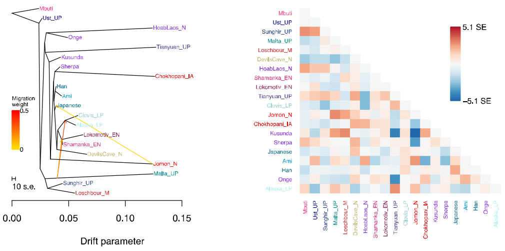

m = 4 , 98.65 % variance explained, ll = 1325.77

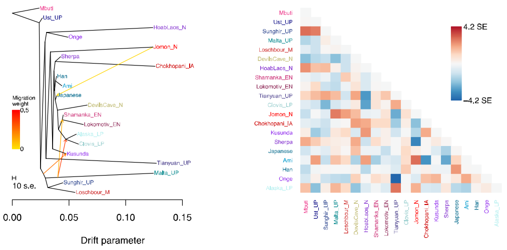

m = 5 , 98.81 % variance explained, ll = 1340.34

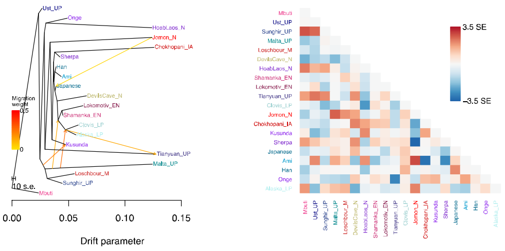

m = 6 , 98.96 % variance explained, ll = 1367.79

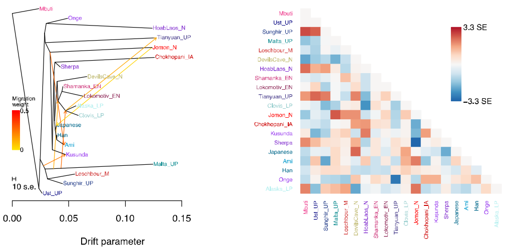

m = 7 , 99.1 % variance explained, ll = 1382.91

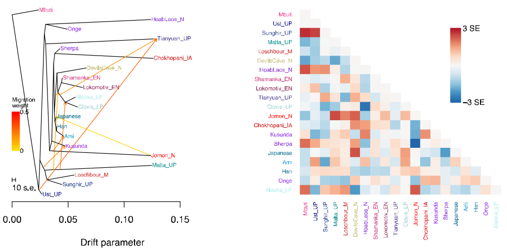

m = 8 , 99.16 % variance explained, ll = 1391.46

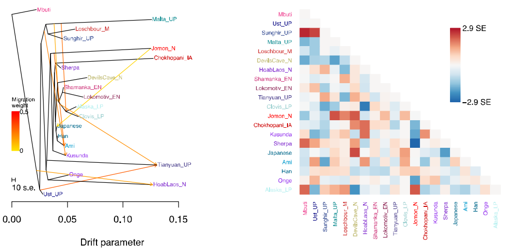

Supplementary Figure 8: TreeMix analysis including Southeast, East and Northeast Asians, Native Americans, and ancient Eurasians, with different migration factors (*m* = 1, 2, 3, 4, 5, 6, 7, 8). The scale bar shows the average standard error (SE) of the entries in the covariance matrix. Coloured arrows represent the migration pathways and signals of admixture among all datasets. The migration weight represents the fraction of ancestry derived from the migration edge.

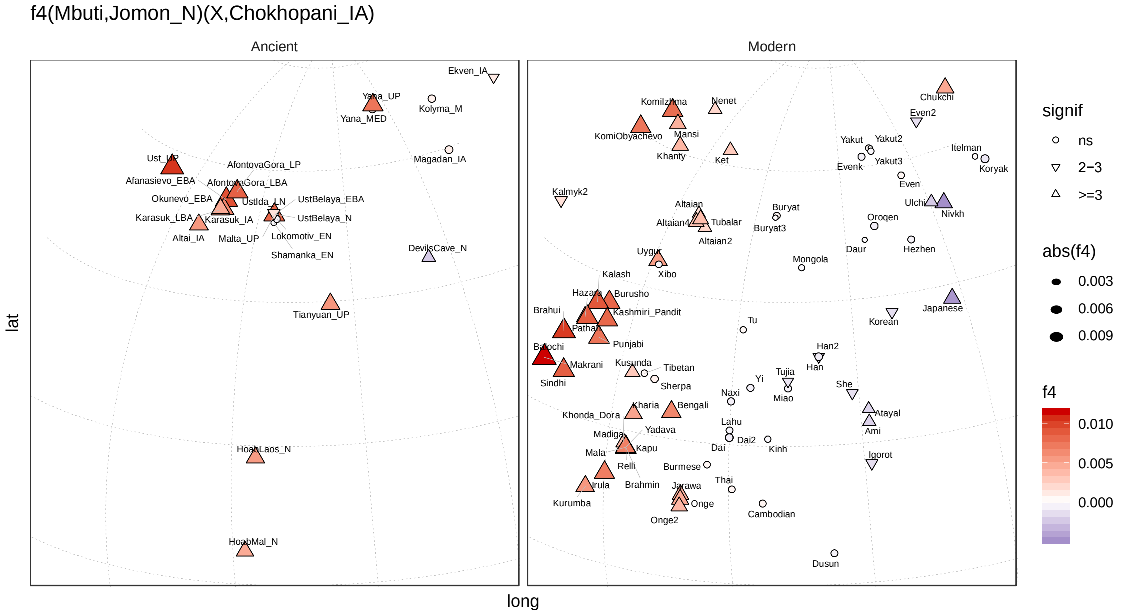

Supplementary Figure 9: Heatmap of f_4 statistics comparing eastern Eurasian populations to IK002. Heatmaps of f_4(Mbuti, IK002; *X*, Chokhopani), where X are (**a**) ancient and (**b**) modern East Eurasian populations.

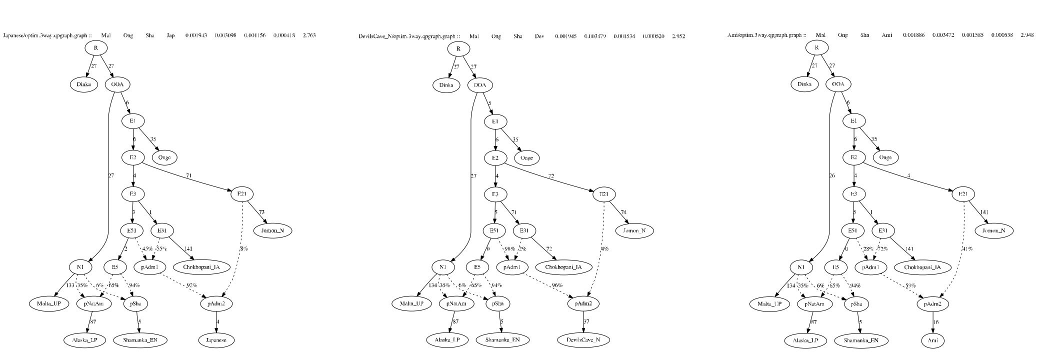

Supplementary Figure 10: the estimated admixture models of each of three target populations (Japanese, Devils Cave and Ami) using qpGraph based on three-way mixtures of early (IK002) and later (Chokhopani, Shamanka) dispersal lineages.

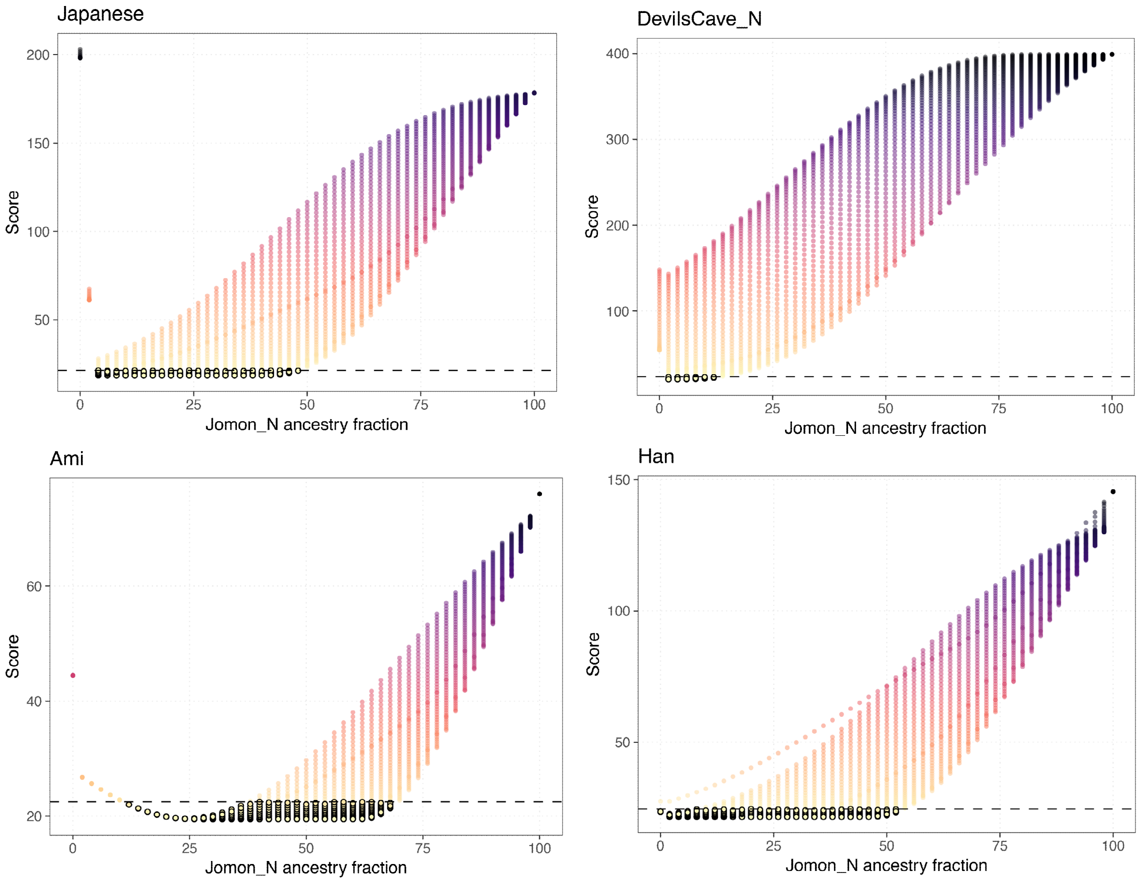

Supplementary Figure 11: Jomon-related ancestry in selected population. For each target population (indicated in panel title), we plot the qpGraph fit score as a function of IK002-related ancestry. Each point represents a single fit from a grid search in a fixed three-way admixture topology (Supplementary Figure 10). The search was conducted in a 100 x 100 grid of admixture contributions from the Jomon_N lineage (node E21 in Fig. S10) and from the Chokhopani_IA lineage (node E31 in Fig. S10). Symbol colors indicate the fit score, with cooler colors representing worse scores. Symbols with black outlines indicate parameter values that yield a fit within three units of the best score (dashed line). Accepted fits generally cover a

wide range of admixture fractions, however only for Han a model without Jomon_N contribution provides an accepted fit.

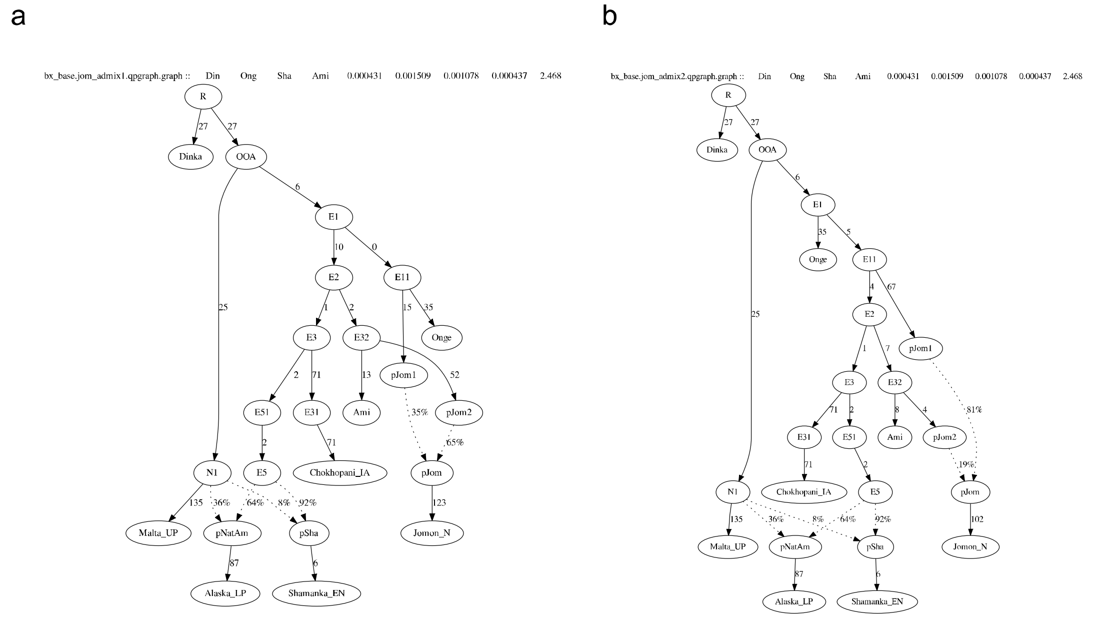

Supplementary Figure 12: the estimated admixture models of the Jomon-related ancestry using qpGraph.

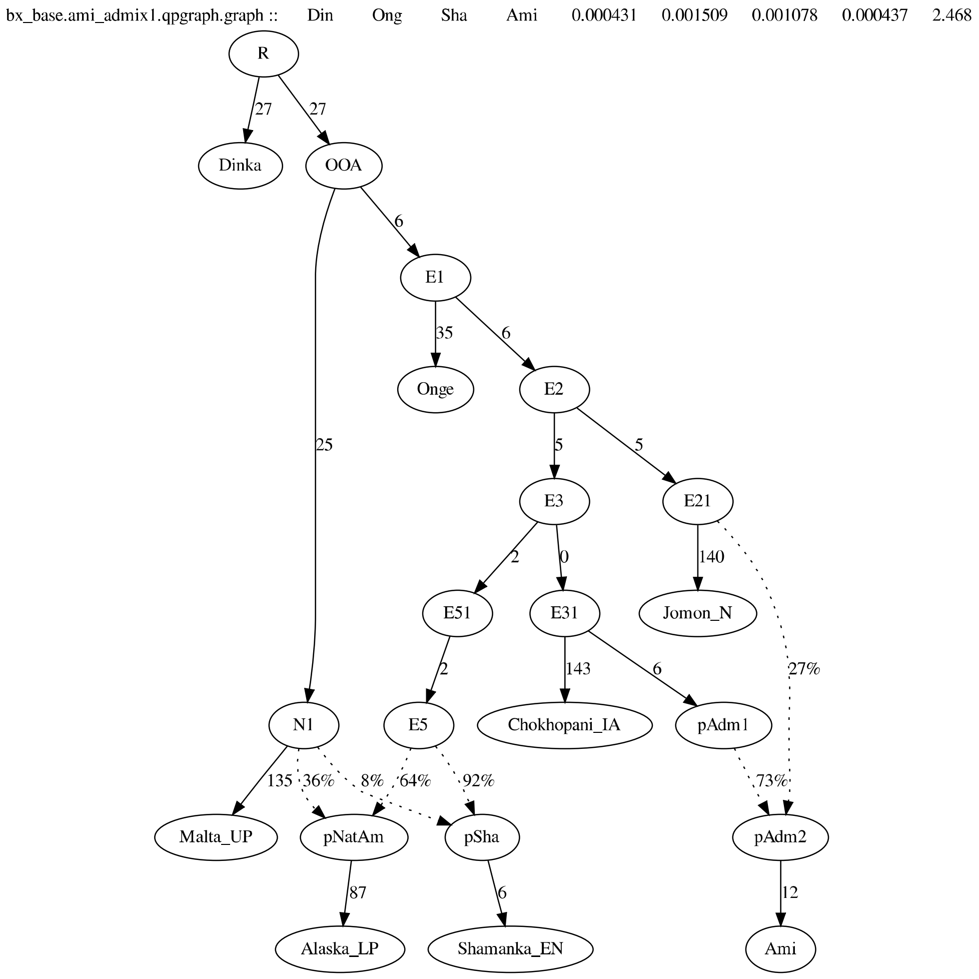

Supplementary Figure 13: the estimated admixture model of the Ami-related ancestry using qpGraph.

Supplementary Discussion

Geological history, archeological/anthropological records, and history of studies on peopling history in the Japanese archipelago

The trace of *Homo sapiens* activity in Japan appears around 38 kya when the Japanese archipelago was connected with the Eurasian continent (Maritime Province of Siberia) by land through the current Sakhalin and Hokkaido islands (so called, the Paleo-SHK peninsula). Because the Blakiston line (the Strait of Tsugaru) between the Paleo-SHK peninsula island and the Paleo-Honshu island (current main-island) was much shorter than present state (Supplementary Figure 1), it was likely to cross easily the narrow strait for the migrants from the East Eurasia continent (Hokkaido Route). At the time, the Kyushu island was not connected with the Korean peninsula. In the coldest period of Last Glacial Stage (LGS: ~20 kya), the sea level went down, and the Straits of Korean and Tsushima were considerably narrowed (Supplementary Figure 1). Then, another route was opened for the migrants into the northern part of Kyushu island through the Korean peninsula (Tsushima Route). Meanwhile, the Ryukyu islands were not connected between the Taiwan island and the Kyushu island at the time (Supplementary Figure 1), though the Taiwan island was connected with the southern China. If migrants used boats, they could have reached the southern part of Kyushu island (Okinawa Route). These three were possible routes in the Upper Paleolithic period from East Eurasia into the region of the current Japanese archipelago[1].

Few skeletal remains reliable have not been excavated from the Paleolithic sites in the Japanese archipelago, but from the Ryukyu islands[2,3]. Sato *et al*. (2015) suggested, however, that no genetic contribution from such Upper Paleolithic people directly to the present-day inhabitants was detected based on modern genomic data from the Ryukyu islands[4]. After the end of LGS, the Japanese archipelago has been separated completely from the Eurasian continent around 15 - 10 kya; the Upper Paleolithic people must have been isolated genetically from the other East Eurasian populations until the agricultural migrants come from the continent around 3,000 – 2,000 years ago[5], which is so called the Yayoi period).

In contrast to the Paleolithic period, a considerable number of skeletal remains have been excavated from the main island (Honshu) in the Jomon period, starts around 16.0 kya in the appearance of the Jomon earthenware and ends around 3 - 2.5 kya when the "end" is defined by staring rice cultivation. The Jomon period corresponds to the Neolithic period in Europe, but

the Jomon people did not have large-scale agriculture, but lived in hunting-fishing-gathering. In the Ikawazu Shell-mound site, the people did not start rice cultivation yet, and had typical Jomon culture. Because the cultural continuity has been detected from the Upper Paleolithic to the Jomon, it is usually thought that the Jomon people are direct descendants of the Upper Paleolithic people. In the eastern part of Eurasian continent, frequent migrations and admixture occurred during the Neolithic period. But, in the Japanese archipelago, the Jomon people were isolated because of the geographical condition, and must have had very less influence of such genetic disturbance. Therefore, the Jomon genome has been thought to be not changed a lot from the Upper Paleolithic genome.

A previous archaeological study hypothesized that ancient Austronesians in Taiwan and Philippine migrated into the southern Ryukyu islands in between 4.8 and 4.0 kya[6]. However, a recent study provides strong evidence on the cultural continuity in the Ryukyu islands from the early to middle Holocene[7]. Our admixture graph in Fig.S10 supports a model whereby the Ami originated from the admixture between the Jomon and the other lineage who had both Shamanka- and Chokhopani-related ancestries, with a relatively low fitting score (Z=2.9). This fitting was improved if we assumed no admixture in this second source population (Z=2.5 in Supplementary Figure 13), which implies very little impact of the Austronesian migration on the genetic makeup of Jomon.

Reference

1. Kaifu, Y., Izuho, M. & Goebel, T. Modern human dispersal and behavior in Paleolithic Asia. in *Emergence and diversity of modern human behavior in Paleolithic Asia* 535–566 (Texas A & M University Press College Station, 2015).
2. Nakagawa, R. *et al.* Pleistocene human remains from Shiraho-Saonetabaru Cave on Ishigaki Island, Okinawa, Japan, and their radiocarbon dating. *Anthropol. Sci.* **118**, 173–183 (2010).
3. Baba, H. & Narasaki, S. Minatogawa Man, the oldest type of modern Homo sapiens in East Asia. *The Quaternary Research (Daiyonki-Kenkyu)* **30**, 221–230 (1991).
4. Sato, T. *et al*. Genome-wide SNP analysis reveals population structure and demographic history of the ryukyu islanders in the southern part of the Japanese archipelago. *Mol. Biol. Evol*. **31**, 2929–2940 (2014).
5. Mizoguchi, K. *The Archaeology of Japan: From the Earliest Rice Farming Villages to the*

Rise of the State. (Cambridge University Press, 2013).
6. Summerhayes, R. G. & Anderson, A. An austronesian presence in southern Japan: early occupation in the Yaeyama islands. *Bulletin of the Indo-Pacific Prehistory Association* **29**, 76–914 (2009).
7. Yamagiwa, K. *et al*. A possible new oldest pottery group in the Southern Ryukyu Islands, Japan: Comparative analysis of elemental components of potsherds from the Shiraho-Saonetabaru Cave Site. *Journal of Archaeological Science: Reports* **26**, 101879 (2019).
