# Supplementary material for: Ancient Jomon genome sequence analysis sheds light on migration patterns of early East Asian populations
Source: Commun Biol. 2020 Aug 25;3:437. doi: 10.1038/s42003-020-01162-2 (PMC7447786; doi:10.1038/s42003-020-01162-2)
Supplement: Supplementary file 2 — Description of Additional Supplementary Files [file 42003_2020_1162_MOESM2_ESM.pdf]

Supplementary Data 1: Endogenous human DNA content of ancient individuals in prescreening with Miseq.

Supplementary Data 2: Analysis chart of sample ID, position, protocol and laboratory.

Supplementary Data 3: f4 statistics among X, IK002 and Hokkaido Jomons with Mbuti as the outgroup.

Supplementary Data 4: Outfroup f_3 statistics among X, IK002 with Mbuti as the outgroup.

Supplementary Data 5: Estimation of generation time after admixture of Japanese, Ulchi and Ainu using ALDER with two source of ancestry populations (mainland East Asians (Han, Ami, Korean, Devils cave) and Jomon).

Supplementary Data 6: *D* statistics among MA-1, X(the eastern Eurasians) and Ami with Mbuti as the outgroup.

Supplementary Data 7: *D* statistics among X(the eastern Eurasians), MA-1 and Ami with Mbuti as the outgroup.

Supplementary Data 8: *D* statistics among Ami, X(the eastern Eurasians) and MA-1 with Mbuti as the outgroup.

Supplementary Data 9: f_4 statistics among Jomon, Ami and Onge with Mbuti as the outgroup.

Supplementary Data 10: f_4 statistics among Onge, X, IK002 with Mbuti as the outgroup.
