# Supplementary material for: Ancient Jomon genome sequence analysis sheds light on migration patterns of early East Asian populations
Source: Commun Biol. 2020 Aug 25;3:437. doi: 10.1038/s42003-020-01162-2 (PMC7447786; doi:10.1038/s42003-020-01162-2)
Supplement: Supplementary file 13 — Reporting Summary [file 42003_2020_1162_MOESM13_ESM.pdf]

nature research

Corresponding author(s): *Double-blind peer review submissions: write DBPR and your manuscript number here instead of author names.*

Last updated by author(s): May 29, 2020

Reporting Summary

Nature Research wishes to improve the reproducibility of the work that we publish. This form provides structure for consistency and transparency in reporting. For further information on Nature Research policies, see our Editorial Policies and the Editorial Policy Checklist.

Statistics

For all statistical analyses, confirm that the following items are present in the figure legend, table legend, main text, or Methods section.

n/a	Confirmed	
[ ]	[x]	The exact sample size (n) for each experimental group/condition, given as a discrete number and unit of measurement
[ ]	[x]	A statement on whether measurements were taken from distinct samples or whether the same sample was measured repeatedly
[ ]	[x]	The statistical test(s) used AND whether they are one- or two-sided<br>*Only common tests should be described solely by name; describe more complex techniques in the Methods section.*
[x]	[ ]	A description of all covariates tested
[ ]	[x]	A description of any assumptions or corrections, such as tests of normality and adjustment for multiple comparisons
[ ]	[x]	A full description of the statistical parameters including central tendency (e.g. means) or other basic estimates (e.g. regression coefficient) AND variation (e.g. standard deviation) or associated estimates of uncertainty (e.g. confidence intervals)
[ ]	[x]	For null hypothesis testing, the test statistic (e.g. F, t, r) with confidence intervals, effect sizes, degrees of freedom and P value noted<br>*Give P values as exact values whenever suitable.*
[x]	[ ]	For Bayesian analysis, information on the choice of priors and Markov chain Monte Carlo settings
[ ]	[x]	For hierarchical and complex designs, identification of the appropriate level for tests and full reporting of outcomes
[x]	[ ]	Estimates of effect sizes (e.g. Cohen's d, Pearson's r), indicating how they were calculated

Our web collection on statistics for biologists contains articles on many of the points above.

Software and code

Policy information about availability of computer code

Data collection	We didn't use the custom code in data collection. We described and cited the methods of data collection in this paper.
Data analysis	We described and cited the details of data analyses in this paper.

For manuscripts utilizing custom algorithms or software that are central to the research but not yet described in published literature, software must be made available to editors and reviewers. We strongly encourage code deposition in a community repository (e.g. GitHub). See the Nature Research guidelines for submitting code & software for further information.

Data

Policy information about availability of data

All manuscripts must include a data availability statement. This statement should provide the following information, where applicable:

- Accession codes, unique identifiers, or web links for publicly available datasets
- A list of figures that have associated raw data
- A description of any restrictions on data availability

This study has been evaluated by the Education Affairs in Tahara city in Aichi Prefecture, Japan. Genomic data are available for download in the DDBJ Sequence Read Archive (DRA) under the accession numbers DRX1892068 and at the ENA (European Nucleotide Archive) with accession number PRJEB26721.

Field-specific reporting

Ecological, evolutionary & environmental sciences study design

All studies must disclose on these points even when the disclosure is negative.

Study description	This paper focused on population genomics for ancient and present-day human.
Research sample	Ancient human remains of Jomon culture in Japanese Archipelago.
Sampling strategy	To obtain the sufficient datasets for genomic analysis, we pre-screened the sample quality from each remains. After pre-screening, we choose the best one within all samples.
Data collection	NGS data was generated by Miseq and Hiseq in Kitasato University and Copenhagen University.
Timing and spatial scale	The excavations of Hobi and Ikawazu sites were carried out from 2010, the human remains from Ikawazu, Hobi and Hegi sites were sampled from 2012 to 2015.
Data exclusions	No data exclusions
Reproducibility	We carried out double-checking the results of genomic datasets from the Ikawazu Jomon remain between Kitasato University and Copenhagen University.
Randomization	This study don't need randomization because this study focuses on specific human remains.
Blinding	This study don't need blinding because this study focuses on specific human remains.

Did the study involve field work? ☐ Yes ☒ No

Reporting for specific materials, systems and methods

We require information from authors about some types of materials, experimental systems and methods used in many studies. Here, indicate whether each material, system or method listed is relevant to your study. If you are not sure if a list item applies to your research, read the appropriate section before selecting a response.

Materials & experimental systems

n/a	Involved in the study	
☒	☐	Antibodies
☒	☐	Eukaryotic cell lines
☐	☒	Palaeontology and archaeology
☒	☐	Animals and other organisms
☒	☐	Human research participants
☒	☐	Clinical data
☒	☐	Dual use research of concern

Methods

n/a	Involved in the study	
☒	☐	ChIP-seq
☒	☐	Flow cytometry
☒	☐	MRI-based neuroimaging

Palaeontology and Archaeology

Specimen provenance	The Education Affairs in Tahara city in Aichi Prefecture, Japan.
Specimen deposition	The specimen were stored in the Education Affairs in Tahara city in Aichi Prefecture and Tokyo University, Japan.
Dating methods	Radiocarbon dating was carried out by the compact AMS (CAMS) in University Museum in Tokyo University, Japan.<br>Analysis ID (TKA) of AMS was attached by the AMS lab in University Museum in Tokyo University, Japan.

☒ Tick this box to confirm that the raw and calibrated dates are available in the paper or in Supplementary Information.

Ethics oversight	The Education Affairs in Tahara city in Aichi Prefecture, Japan.

Note that full information on the approval of the study protocol must also be provided in the manuscript.
